# New Concepts in Breast Cancer Emerge from Analyzing Clinical Data Using Numerical Algorithms

**DOI:** 10.3390/ijerph6010347

**Published:** 2009-01-20

**Authors:** Michael Retsky

**Affiliations:** Children’s Hospital and Harvard Medical School, Karp Family Laboratories, 300 Longwood Avenue, Boston, MA 02115, USA; E-mail: michael.retsky@childrens.harvard.edu

**Keywords:** Breast cancer, quasi-stable dormancy, surgery-induced angiogenesis, dormancy preservation therapy

## Abstract

A small international group has recently challenged fundamental concepts in breast cancer. As a guiding principle in therapy, it has long been assumed that breast cancer growth is continuous. However, this group suggests tumor growth commonly includes extended periods of quasi-stable dormancy. Furthermore, surgery to remove the primary tumor often awakens distant dormant micrometastases. Accordingly, over half of all relapses in breast cancer are accelerated in this manner. This paper describes how a numerical algorithm was used to come to these conclusions. Based on these findings, a dormancy preservation therapy is proposed.

## 1. Introduction: Breast Cancer Research Challenges Conventional Theories

Over the past 11 years, my colleagues and I have published a series of over 30 papers that have challenged well-established theories in breast cancer. In collaboration with Romano Demicheli, MD, PhD, of Milan National Cancer Institute, we proposed in 1997 that in order to fit relapse data, breast cancer growth includes periods of temporary dormancy [1, 2]. Furthermore, we proposed that surgical removal of a primary tumor kick-starts growth of dormant distant single malignant cells and avascular micrometastases. These are large effects. Over half of all breast cancer relapses seem to be accelerated due to these mechanisms. In additional collaboration since 2000 with William Hrushesky, MD, now at University of South Carolina, it has been shown that the earliest relapses (i.e., within 10 months of surgery for patients untreated with adjuvant chemotherapy) occur in 20% of premenopausal-node-positive patients [3–5].

These theories were proposed to explain a bimodal relapse pattern which was initially reported in two databases and has now been identified in 13 independent databases [6–17]. This bimodal pattern strongly implies that there is more than one mode of relapse. Our theories propose that the first peak of relapses are events that are iatrogenic, i.e., triggered by surgery and that the second relapse peak represents the natural history of the disease.

**Figure 1:**
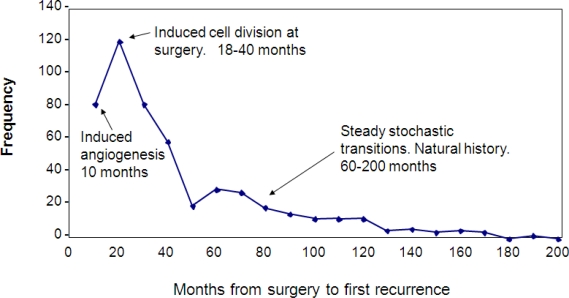
Data from Milan are shown in raw form as number of relapse events in 10-month wide bins. Also indicated are the various modes of relapse that are predicted by the computer simulation.

Figure 1 shows the Milan data, together with our explanation of the various relapse modes. The first peak is clearly dominant and includes two previously unreported relapse modes. The earliest relapses (within 10 months of surgery) are avascular micrometastases that are induced into angiogenesis by surgery. The relapse events in the remainder of the first peak are the result of single dormant malignant cells that are induced into division by surgery and then pass through angiogenesis stochastically. Mechanisms proposed for this first peak are growth factors produced in response to surgical wounding and also reduction of antiangiogenic factors due to removal of the primary tumor. After a nadir at 50 months, the second peak that extends out to 200 months is seen. This second peak is the natural history of the disease. Events in the second peak are the result of stochastic transitions from one state to the next in the growth progression as proposed. The top of the second peak marks the point at which the benefits of surgical removal of the primary tumor are first seen. Until that time, surgery has produced accelerated relapses.

**Figure 2:**
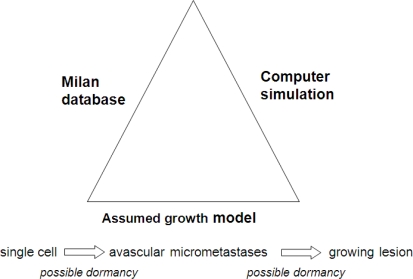
Overview of the algorithmic approach to the analysis of the Milan database.

**Figure 3:**
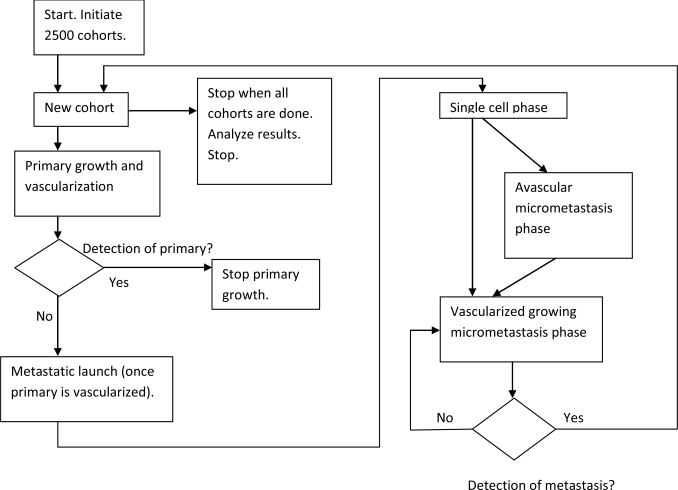
The computer program that was used to determine numerical values of growth rates and transitions from dormancy is shown in flow chart form.

The outlines of our algorithmic approach are shown in Figures 2 and 3. The three necessary ingredients needed to perform an algorithmic study are shown symbolically in Figure 2. First there are the clinical data from the Milan National Cancer Institute on follow-up of 1,173 patients after removal of the primary tumor. The growth model chosen allows the possibility, but does not insist that tumor growth can be interrupted for variable periods of time at the single cell level and also before angiogenesis occurs. This growth model is the sum and substance of the assumptions in this project. The third ingredient is a stochastic computer simulation.

**Figure 4:**
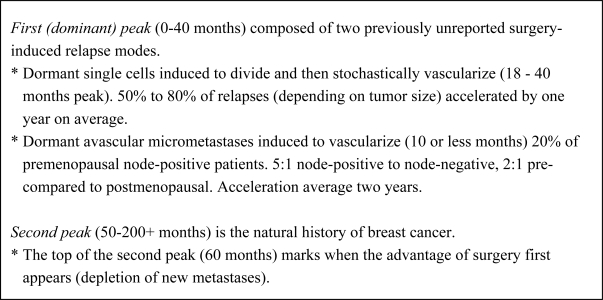
Results of computer simulation of Milan database.

The computer program used to determine numerical values of growth rates and transitions from state to state is shown as a flow chart in Figure 3. It is meant to simulate the full history of early stage breast cancer for a large cohort of subjects. In the simulation, breast cancer starts with one malignant cell in the primary tumor. The primary is allowed to grow. Once the primary has exceeded approximately 1 mm diameter and until it is detected and removed, metastatic cells are shed into distant sites where they lodge and grow until one is detected as a metastatic relapse. The shedding process occurs most strongly for patients with a high risk of relapse such as having positive lymph nodes. As the primary tumor gets larger, more metastatic cells are disseminated. Thus the risk of relapse also increases with the size of the primary tumor at detection, consistent with clinical data. For some patients, there are no metastatic cells shed and obviously they never relapse. Dormancy of micrometastases is allowed but not required at the single cell state and also prior to angiogenesis. Metastatic shedding and exits from dormancy are simulated as stochastic events. This process is conducted for 2,500 separate individual cohorts. Using a population this size produces reasonable repeatability of results. Not coincidentally, large clinical trials have approximately that number of subjects. The single most important result was that transitions needed to be temporarily augmented at the time of surgery in order to explain the first peak in the bimodal data. Results of using this algorithm are summarized in Figure 4.

We reported in 2001 that surgery-induced angiogenesis (as first proposed by Judah Folkman 30 years ago, in order for a tumor to grow beyond a mm or so, a blood supply needs to be provided by the host; this process is called angiogenesis.) quantitatively explains the so-called mammography paradox for women aged 40–49. That is, when mammography was first tested in randomized controlled clinical trials, women aged 50–59 showed an early appearing 20–30% mortality benefit. However, when this was evaluated in women aged 40–49, there was a counterintuitive early *disadvantage* that eventually turned into the expected advantage after 6–8 years. Most of the patients in these early trials were not treated with adjuvant therapy. The 2001 paper quantitatively showed how surgery-induced angiogenesis based on the Milan data agrees with the timing and magnitude of the mammography data. The most recent trial of mammography for women aged 40–49 showed a non-significant advantage at 10 years into the trial [18] whereas there is significant advantage for women aged 50–59. So even with modern adjuvant therapy, early detection apparently works better for women aged 50–59 than it does for women aged 40–49. Women are now screened starting at age 40 in the US, while in most of Europe it starts at 50.

We further proposed in 2004 that surgery-induced angiogenesis explains why adjuvant chemotherapy works the best by far for premenopausal-node-positive patients. NIH Consensus reports both in 1980 and in 1985 suggest that adjuvant chemotherapy is useful for premenopausal patients with positive nodes. According to our theory, sudden induced tumor growth after surgery results in a chemosensitive period just at the time when adjuvant chemotherapy was empirically found to be most effective. Two papers from 2004 on this subject have been downloaded over 25,000 times [19, 20]. Co-authors in one of these papers are Gianni Bonadonna, who was a key pioneer in using chemotherapy 30 years ago, and Judah Folkman, who founded the field of tumor angiogenesis.

A recent paper with surgeon Michael Baum reported that it was known 2000 years ago by premodern “chirurgeons” that surgery to remove a tumor could speed up relapses [21]. Our most recent work includes Nigerian surgeon Isaac Gukas, MD, PhD, as another collaborator. We suggest that the excess breast cancer mortality of African-Americans can partially be explained by surgery-induced angiogenesis, since the average age of diagnosis of African-Americans is 46 years compared to 57 years for European-Americans [22]. This excess first appeared in the 1970s when mammography began. So in view of our explanation of why early detection works better for postmenopausal women than it does for premenopausal women, and that black breast cancer is usually premenopausal while white breast cancer is postmenopausal, it should be no surprise that an excess in breast cancer mortality began when mammography was introduced.

The really unusual part of this story is that I have no formal background in biology or medicine. Rather, my background is in experimental physics. (Dr. Demicheli also has a background in physics.) How I became a breast cancer researcher and how the use of traditional tools from physics including numerical algorithms came to play a key role is a very interesting story and I enjoy telling it. I do not know if my unorthodox career pathway can serve as a template for future researchers, but let me document this story and perhaps some scientist in the future will be inspired to do something similar or at least know what to expect.

## 2. What Are the Skills and Research Culture of Experimental Physics That Might Apply to Cancer Research?

For many scientists who chose to pursue an experimental science profession, their PhD thesis project was a formative educational activity where a full experiment was done, from conception to publication of final results of original research, under the watchful eye of a knowledgeable and experienced professor. The mentor’s style and strategy usually imprint on the student and form the basis of future research. Thus, to best describe my research skills and methodology, I am going to discuss my doctoral thesis project at the University of Chicago in the Physics Department under the guidance of Prof. Albert Crewe.

Prof. Crewe originally worked with particle accelerators in the UK. He was renowned for developing a method and building equipment to extract proton beams from the accelerator for use in the experiments. His scientific strength was best demonstrated when he was given a new problem to solve. It could be almost anything in the physical sciences. He would sit at a desk with a few sheets of high-quality white paper and a fountain pen and calculate the likely result of various possible ideas that might address the problem. With his excellent knowledge of basic physics and material science, he could calculate almost anything within a factor of 2 in a very short time. Physics is a science with a strong computational basis. There are a handful of very basic equations and a mathematical hierarchy that allow almost everything to be calculated from fundamental principles. Most physicists could calculate various effects quite well in their field given enough time, access to libraries and computers. Crewe could do it without those tools. He could discard inferior ideas and identify good ideas rapidly and with reasonable accuracy.

Once in an airplane, he began to think about electron microscopes. From a scientific aspect, that was not too far from particle accelerators. On that journey, he invented a new method of electron microscopy which might image and identify single atoms in DNA, so that perhaps the biological code could be read on individual DNA molecules. Electron microscopy had reached an impasse in resolution that could be traced in large measure to the electron sources used which were very hot pieces of tungsten that essentially boiled off electrons that could then be accelerated and focused. The main difficulty was that high aberrations resulted from the spread in energy of these hot electrons. Crewe deduced that room temperature electrons would be needed. One way to produce a beam of electrons from a cool substrate would be to chemically etch thin wires to form very sharp points. Then with a high electric field, cold electrons could be pulled off and used to form a beam. This source would be bright and could conceptually be focused to a very small size since the source has relatively small size.

The problem was that a whole new technology was needed to learn how to reliably make these new, very delicate electron sources, learn how to use them and to build a microscope to form a very sharp focus. Before I got accepted into the Crewe Laboratory, they had already built a microscope that operated at 30 kV and produced some famous pictures of what they thought were single atoms of uranium [23]. We later determined that these were clumps of atoms rather than single ones. My project was to build the second-generation microscope that supposedly would solve many of the problems found in the original design and operated at higher voltage of 50 kV. Theoretically, resolution improved with higher voltage but design and high voltage breakdown problems were harder.

As per Crewe’s style, designs were done based on calculations completed before starting any machine shop fabrication. Everything possible was calculated beforehand. Nothing was done by a trial and error or “seat-of-the-pants” method. My main contribution to the technology was in building the highly stable current supplies for the magnetic lenses and high voltage supplies [24]. My supplies were stable to one part per million per hour, which was 10-fold better than anything previously done. This stability was needed for the unprecedented resolution for which we were aiming.

I eventually got the 50 kV microscope to the highest resolution possible for that particular design which was 0.24 nm and ultimately limited by the Uncertainty Principle. Using the microscope, I could reliably distinguish a single silver atom from a single uranium or mercury atom but could not distinguish a mercury atom from a uranium atom [25]. Crewe’s original motivation—to read the DNA code—ultimately did not work out because the electron beam caused too much damage to the DNA molecule [26]. Experimental science even when done properly sometimes does not work as planned.

## 3. The Scientific Method and Models As Used in Physics

It is often taken for granted, but we owe much of the enormous advance in scientific knowledge gained in the past few centuries to the careful and rigorous application of the scientific method. Before implementation of this method, for their own reasons, religious or state leaders could arbitrarily proclaim what was and what was not scientific truth. As an example of how this can interfere with science, consider the famous Galileo episode.

In 1610, the Italian Galileo was the first to observe the moons of Jupiter and the phases of Venus with an early telescope. On the basis of these as well as observations of tides, he promoted the Copernicus theory that the earth revolved around the sun rather than the accepted Ptolemaic theory that planets and the sun circled a fixed earth. He was sentenced to imprisonment in 1633 (later commuted to house arrest) for heresy. The center of science then shifted from Italy to Northern Europe and the British Isles. (Newton did his work in the late 1600s.)

Comparing that disruptive change with the more orderly abandonment of the ether theory as the medium in which light propagated, this was as important as the Galileo incident. Since light is a wave and all observed waves produced oscillations in some media, it was held that there must be a medium called ether in free space. The vibrations of ether would allow light to travel. Michelson and Morley (1887) showed that the speed of light was invariant of the direction of light propagation relative to the direction of earth’s travel through space. The experimental uncertainty was 10 km/s and the earth’s speed in its orbit is roughly 30 km/s. It was difficult to reconcile this experiment with the ether theory. The theorists dragged their heels trying to reinterpret these data according to their ether theory but the experimentalists eventually prevailed. This paved the way for Einstein to propose his theory of special relativity in 1905 which was dependent on the invariance of the speed of light. This theory is considered to be the beginning of modern physics.

In its most basic form, the scientific method is a continual comparison of theory and experiment. Theoreticians propose theories that explain existing data and experimentalists perform experiments to test existing theories. A good theory must explain a wide variety of data based on only a few arbitrary assumptions and it must be testable. Of utmost importance in this relationship, when there is disagreement between experimental results and theoretical predictions, it is theory that must yield. It does not matter whose theory it is or how long it has been accepted. When new data disagree with prevailing theory, the theory must be called into question. New theories are then proposed to explain these new data. In turn, further experiments are done to test the new theories. This process continues until theories and experiments are in accord. At that time, the theory is accepted as a valid explanation of the phenomenon.

While we would like to think that it is, this process is not as rigorously practiced in medicine. The problem stems in part from the lack of freedom, due to ethical restrictions, to perform key experiments to test a theory. An additional problem is that in some chronic diseases such as breast cancer, the long term of the disease process is daunting. Consider the practical problems in breast cancer research. Researchers and physicians are trying to formulate theories and empirically optimize therapies for a disease that takes 15 or more years to run its course. Imagine trying to tune the engine of your vintage sports car if there were a 15-year period between adjusting the carburetor and waiting for the engine to respond.

In cases when experiments are impractical or impossible, researchers often resort to models. Models are acknowledged imperfect representations of the system that you want to study but for one reason or another cannot be freely experimented upon. Physicists are accustomed to this, since they have long dealt with systems that are too distant, too hot, too cold, too fast, too long ago, too small, etc. They have developed very sophisticated computational models that can be used to develop theories and to compare theoretical and experimental results. The sophistication of modern mathematical or computer models can be so profound that they are sometimes considered to be equivalent to an experiment. Imagine that they know what happens in a supernova stellar explosion or, in a more sinister application, how to design an atomic weapon by using computer models. A problem that occurs while utilizing models is that the differences between the model and the system to be represented should be quite well understood and acceptable. Otherwise, results of the model may be misleading and the actual system may not respond as predicted by the model.

## 4. Models As Used in Cancer Research

There are two important cancer models that I want to discuss—the experimental animal model of tumor growth and the Gompertzian equation used as a tumor growth law.

**Figure 5:**
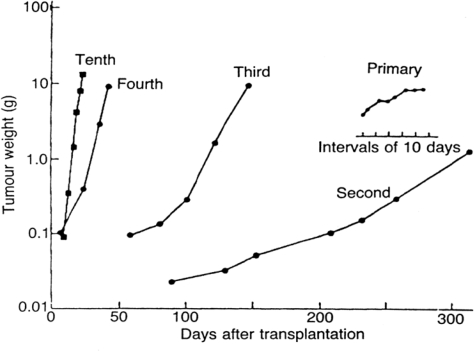
Data from Steel [27] are shown as modified. This demonstrates how a multipassaged animal model tumor originally showing erratic growth becomes a reproducible and rapidly growing tool suitable for experimental use.

The common model of cancer in the laboratory is the animal model. As shown in Figure 5, this tumor starts as an erratic spontaneous tumor in an animal or human and is passaged many times usually through immune compromised animals until by selection for the most robust and rapidly dividing cells, it is a reliable and reproducible entity suitable for experimentation [27]. The process of developing such a model produces a tumor that reliably doubles in volume in a few days and does not exhibit dormancy. That is approximately as fast as a cell can divide. Since a detectable size 1 cc tumor consists of a billion cells, more or less, and 1 billion is approximately 2^30^, it takes only 30 doublings or a few months to grow a tumor from one cell to a detectable tumor. Thus, an experiment can be done in a reasonable time frame for a research grant and one can reliably compare results from one laboratory to another anywhere in the world. All cells are constantly dividing in such a tumor. However, a human breast cancer has average doubling times of approximately 100 days, i.e., roughly two orders of magnitude slower than animal models. There are also periods of time, sometimes years in length, when a breast tumor does not increase in size. How else can we explain relapses that can occur 10 or 20 years after primary removal and differ little from earlier relapses other than when they occur? Or, indeed, how else can one explain all the reports of dormancy especially in breast cancer [28–40]? Thus, the much-used animal model is quite different from the breast cancer system that it is meant to represent. This difference has not been fully appreciated. In fact, it has only very recently been acknowledged that dormancy exists in cancer and needs to be considered. This was recently highlighted in a *Cell Cycle* editorial as the dormancy “problem” [41]. As an indication of the potential importance, it has been experimentally shown that dormant cancer cells are highly refractory to chemotherapy and are fully viable to grow and produce a tumor afterwards as if no chemotherapy was administered [42].

**Figure 6:**
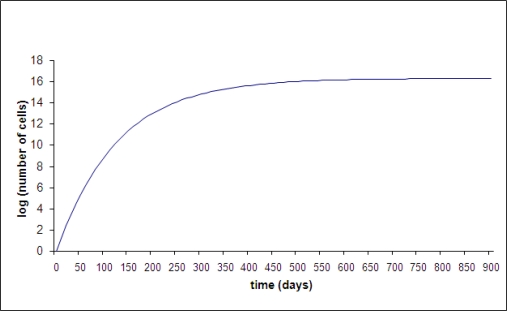
Gompertzian growth shown as log tumor burden (as represented by number of cells) vs. time (days).

The other commonly used cancer model is the theory that breast cancer grows according to the Gompertz equation [43–47]. Gompertz was a 19^th^ century actuarial scientist who proposed his equation as a general description of population growth. This growth starts exponentially (constant doubling time) and gradually slows down until it ultimately reaches a limiting plateau, as shown in Figure 6. This is related to the Malthusian concept that the population of a city or state is ultimately limited by its food supply and ability to dispose of waste. Gompertzian growth is continuous, i.e., it cannot grow, stop, and then grow again. Gompertzian kinetics has played an important historical role in cancer chemotherapy and is still often cited. According to this theory, at the time of diagnosis of primary breast cancer, metastatic disease is as small as it ever will be in the clinical setting—thus growing as fast and as chemosensitive as possible. Therefore, the optimal strategy for adjuvant chemotherapy is to use very intensive therapy as soon as possible after surgery and then hope for the best. This idea is traceable to the classical experimental work of Skipper and Schabel in the 1970s [48]. Their work was done using multipassaged animal models with 1 or 2 day doubling times. They could cure animals if and only if all cancer cells were eradicated. Their papers included terms such as LD_50_ and LD_90_ to indicate that some chemotherapy protocols were lethally toxic to 50% or 90% of animals. The high success of using chemotherapy to treat animal model tumors has translated into only modest benefit in clinical breast cancer and accompanied by significant toxicity. Perhaps this is a result of the large differences in growth rates between multipassaged animal tumors and breast cancer and the reliance on Gompertzian kinetics to guide therapy.

I have published my findings that the entire experimental basis for Gompertzian kinetics lies in the 1960s era Laird papers [49–54]. Laird measured growth of “19 examples of 12 different tumors of the rat, mouse, and rabbit” and concluded: “The pattern of growth defined by the Gompertz equation appears to be a general biological characteristic of tumor growth.” That is a far reaching statement based on only 18 rodents and one rabbit.

Using a least square method, Laird fit a Gompertzian equation to each individual tumor. Then Laird compared the best fit Gompertzian curve with a simple exponential curve and concluded that the Gompertzian curve fit these data better than the exponential curve. However Laird used numerical parameters in the exponential equation that were taken from the best fit Gompertzian equation. The proper comparison would be a best fit Gompertzian to a separately best fit exponential.

As a numerical example of this, for one particular tumor Laird fit W=W_0_ * exp [(0.788/0.142) * (1 – exp (−0.142 t))]. That Gompertzian expression reasonably well fit those data. Then expanding [1 – exp (−0.142 t)] in a Taylor series as [1 – 1 + 0.142 t - …] or 0.142 t (taking the first non-zero term), Laird concluded that the exponential curve to compare to the best fit Gompertzian fit is exp [0.788 t]. As no surprise, the pure exponential curve was accurate at t = 0 but rapidly became far larger than the best fit Gompertzian. This flawed analysis was conducted for each of the 19 tumors Laird reported. Remarkably, Gompertzian kinetics as a valid description of breast cancer growth has been virtually unchallenged other than from my own publications. Laird papers have been cited over 1,000 times. Despite that, I came to the conclusion that I was the only person who actually read these papers.

The Gompertzian growth model has long been assumed to describe primary and metastatic breast tumors. The growth starts as exponential (constant doubling time) which would appear as a straight line on this semi-log scale. Gompertzian growth is a damped exponential which means it gradually slows and approaches an asymptotic value. There are two parameters in this function that determine the initial growth rate and the ultimate size (*N* is the number of cells in the tumor). The equation is *N* = exp[(*A/B*)(1 – exp(−*Bt*)]. In Figure 6, *A* was chosen as 0.3 per day and *B* was chosen to be 0.008 per day. Time *t* is expressed in days. At small time, *N* = exp(*At*), so *A* determines the slope in the early exponential phase. The ultimate size asymptotically attainable in Gompertzian growth is exp(*A*/*B*) and in this example it is 1.9 × 10^16^ cells. This value must be larger than 10^12^ cells, the value that is usually taken as a lethal tumor burden, since untreated cancer is presumed to be uniformly lethal. Thus, the two parameters *A* and *B* are not completely independent.

Gompertzian growth does not allow for dormancy. That is, the growth is continuous from a single cell to ultimate conclusion. In addition, it is assumed that all tumors are independent. That is, removal of the primary tumor is assumed to have no bearing on growth of metastatic disease according to this theory. Gompertzian kinetics may well describe the growth of multipassaged animal tumors especially if they are allowed to attain large size. The main drawback in its use to design clinical treatments stems from the unsupported belief that it was explanatory of “all” the natural history of tumors in the subclinical phase and the clinical phase. A situation such as this would never happen in physics. The equivalent in physics would be if no one reviewed and tested Newton’s Laws.

## 5. Unusual Start of Breast Cancer Research Project

After completing the PhD at Chicago, I worked in the electron beam technology field at Zenith Electronics in Chicago and Hewlett-Packard in Colorado Springs. While at Hewlett-Packard, I became interested in cancer research when a good friend’s wife was diagnosed with gastric lymphoma. A small informal research group was formed consisting of myself, Robert Wardwell (the patient’s husband and project organizer), Victor Petrosky, PhD (H-P scientist) and Jack Speer, MD (the medical oncologist treating Wardwell’s wife). We decided to study breast cancer using computer simulation since there was much data available for that disease and powerful H-P computers were available for our use. I did the computer simulation since that was my forte.

We never had any funding and never had a leader, but there was good chemistry within the group. We would meet on Tuesday evenings at 7 p.m. in Speer’s office at Penrose Hospital. It would start with Speer and Petrosky having an argument about some philosophical point from the prior week, we would then discuss our project, and the meeting would end later with Speer telling a joke.

Due to a very strange circumstance, for five years I had an engineering position at H-P that did not require my full attention, allowing me time to read cancer research papers from the Penrose Hospital Library. Rather than from formal classes or attending lectures, I learned about cancer beginning with the original papers. In addition, Speer was an excellent resource. With my strong educational background in basic science, I was able to discern where evidence was strong and where it was not. While evidence-based medicine is now a well established principle, it was not always so and some standard cancer practices were established at an earlier time.

The close involvement with Dr. Speer, who was treating patients daily, kept the group on a clinical perspective. We wanted to design chemotherapy protocols to better treat patients and we wanted to do it right then. (I must note that in addition to the original core group, in later years, other physicians and scientists including David Headley, Douglas Swartzendruber, Paul Bame, Romano Demicheli, Pinuccia Valagussa, Gianni Bonadonna, and William Hrushesky made important contributions.)

What we thought would be a relatively modest but useful project was to build a computer simulation of breast cancer growth and treatment using well-established concepts of tumor growth and response to chemotherapy. This could then be used to study various chemotherapy protocols. Perhaps simple variations could make large differences in outcome. However, early difficulties were encountered when accepted theories of tumor growth could not be reconciled with published clinical data. Deciding that the theory must be revisited, a new theory of intermittent growth was proposed that agreed with these data. The group eventually published two papers [55, 56], proposing that cancer growth includes periods of dormancy rather than the accepted continuous growth Gompertzian kinetics.

Gradually I became more interested in cancer research than electron optics. Cancer research seemed to be in a state of turmoil much like physics was before the 1920s and 1930s when atomic theory and quantum theory were developed. That is, there were many critical observations in cancer research and therapy, but seemingly there were no overarching connections tying one to another. In comparison, current physics research was much more mature, organized, and orderly and seemed far less exciting. In 1987, H-P wanted to downsize and offered voluntary severance packages. I took that and got a position as Research Professor in the department of Biology at the University of Colorado. I also was Visiting Professor alternate weeks for six months in the Department of Medicine at University of Texas, San Antonio, in the late William McGuire’s group. With access to a Cray supercomputer, we tested the accuracy of the computer simulation using UT’s 5,700 patient database and ended up with 3–4% error. A reference laboratory in Stratford, CT, heard about the project and wanted to provide a prognostic report based on the computer simulation free to their tumor marker clients. My wife and I ended up moving to Connecticut to develop and help market the prognostic report. This activity ended in 1994.

## 6. Diagnosed with Stage 3 Colon Cancer and Deciding on an Unorthodox Adjuvant Therapy

This research project became more than just a theory to me when I was diagnosed with stage 3 colon cancer in November 1994. This resulted from a change in medical insurance that triggered a routine physical. Fecal occult blood test (FOBT) came out positive twice and the resulting colonoscopy showed a large sigmoid tumor. I was awake towards the conclusion of this procedure and there was no doubt what was on the monitor. I knew instantly that I had colon cancer. I put everything else on hold and focused all my attention on my disease and how to treat it.

Surgery to remove the primary tumor was routine. Steve Stein—a highly recommended Yale surgeon performed the surgery. Surgery and recovery went well but my long-term prognosis was quite concerning. There were four positive nodes, the tumor had penetrated the muscularis propria and into the pericolonic fat. In the tumor, p53 was mutated and the tumor cells were aneuploid. I looked up relevant papers and determined that my risk of metastatic relapse was 80% without any chemotherapy and about 50% with conventional therapy.

As might be expected, my scientific inclinations were against conventional short course, intensive chemotherapy. The last few sentences in a paper I presented in Switzerland in 1993 are: “Our studies suggest that (chemo) therapy is too ineffective by a factor of 2 to be considered curative. However, if used judiciously, these same drugs may be effective enough to retain tumors at small size. We believe that this is true even considering acquired drug resistance. It is like a boxer going for the decision rather than the knockout [57].”

Thus, I decided against conventional 5FU—leucovorin chemotherapy. Jack Speer suggested to contact Bill Hrushesky who was then in Albany, NY, at the VA Hospital. After discussions with Dr. Hrushesky, I eventually decided to use low-dose infusional-delivered 5FU. This drug has been around for decades and out of patent protection, so it was very inexpensive—actually less costly than sterile water. This particular method of therapy had been used in late-stage disease to extend life but never used in adjuvant therapy—where I planned to use it.

Hrushesky put me on 70% of the usual long-term tolerable dosage of 300 mg/m^2^/day. The drug was given through a port-a-cath via a programmed portable pump that ran from 4 to 10 p.m. every night. During the other 18 hours/day, the pump went into a trickle or keep-vein-open mode [58]. Toxicity profiles are steep for these drugs. Reducing dosage by just 30% essentially eliminated toxicity.

Researching low-dose infusion 5FU, I found it is useful in a wide variety of cancers and does not seem to develop drug resistance even over long-term use. All chemotherapy drugs develop resistance but, at least at that time, it was thought that antiangiogenic drugs never develop resistance. I was beginning to think that the mechanism of action for my therapy might be partially antiangiogenic.

It was quite burdensome to wear the pump all the time. I then read some papers from France saying the optimal time for drug delivery varied somewhat from study to study. I decided that it probably mattered less the exact time of day the drug was used but quite important to do it every day and for a long time. I ended up using it only at night, starting at bedtime (whenever time that was) and running it for 6 hours. I would disconnect the pump every morning and flush the lines. Wearing a loose fitted shirt, the sub-skin port and attached short tubulation were not visible. At night, I reprogrammed the pump and connected it to my port. This made the protocol much easier.

It was a lot of fussing but I stayed on this therapy virtually every night. On the basis of some calculations, I stopped at 2.5 years. The port was in for about 5 years and I never had a problem with it. I attribute that to the skill of surgeon Steve Stein.

While still on therapy, I became part of Judah Folkman’s Lab as Lecturer in Surgery, since my breast cancer research dovetailed with their experimental work showing the importance of tumor dormancy and how angiogenesis plays a role. In December 1996, I talked to Dr. Folkman about my therapy and asked if they ever tested 5FU for antiangiogenic properties. He said they did and found no such effect. I then asked if they tried it at continuous infusion since the half-life of a large single injection of 5FU is about 20 min and any possible antiangiogenic effect would likely be missed. They had only tested 5FU as a large single injection. Dr. Folkman brought Tim Browder in the discussion. Browder was a pediatric medical oncologist who was doing animal experiments on chemotherapy drugs and testing for possible angiogenesis effects. Browder subsequently purchased micro-pumps that could be implanted in mice to treat using continuous infusion. They determined, to some surprise, that continuous infusion of 5FU and other common cytotoxic drugs such as cyclophosphamide were partially antiangiogenic. The important aspect is to use the drug on a continuous or more frequent antiangiogenic schedule instead of the customary high-dose every few weeks. The vasculature responds but apparently recovers in the period between conventional drug applications.

Due to the revolutionary findings, it was difficult to publish the study which was eventually published in April 2000 [59]. This method of therapy is now called “metronomic chemotherapy” and is being tested in many locales. Browder *et al.* has been cited over 500 times.

This information was passed on to a colon cancer patient website long before it was published. It was too important to wait. Dr. Folkman was receiving many calls daily from cancer patients and their physicians. He referred perhaps 100 calls to me. I know many patients have used it for metastatic disease. My therapy is also described in 2000 in a biography of Judah Folkman by Robert Cooke [60]. There are three pages in that excellent book on my medical history.

As of now, no one else to my knowledge has ever been treated with any low-dose long-term drug such as 5FU as an adjuvant therapy—as I used it and think it will be most effective. It is now 14 years since diagnosis. I am NED (no evidence of disease) and well beyond the risk of relapse time for colon cancer. My health is excellent and there were no short or long-term toxicities such as cognitive dysfunction, neuropathy, or heart disease that sometimes occur for ordinary high-dose adjuvant chemotherapy. (As evidence of the lack of cognitive dysfunction, the computer simulation resulting in the 1997 papers was conducted while I was on therapy.) By the way, Mrs. Wardwell is also fine and last I heard she was managing a hospice unit in Colorado Springs.

I tried but was never able to generate any interest to start a trial or even a pilot study of this therapy. A big problem is that there would be no way to recoup the high expenses of pursuing this effect, since the drug is so inexpensive. Even though my case is well-documented, it is impossible to prove that I was not cured by surgery—a 20% likelihood. At any rate, I was the first person treated with antiangiogenic adjuvant chemotherapy.

Judah Folkman has introduced me on more than one occasion as the first human treated with metronomic (adjuvant) chemotherapy. It is ironic, but dormancy, that is so common in breast cancer, is rare in colon cancer. So my original rationale was probably wrong, but the result turned out right for me anyway.

## 7. Reflections and Recent Developments

It is not for everyone but as an intellectual stimulation, I highly recommend a career change after 20 years in one field. For me it has been a tonic. At an age at which most scientists are well past their prime, I have never been more productive.

Among other things, I recently submitted a patent application for a new primary antiangiogenic treatment plan for early stage breast cancer that is non-toxic and should be far more effective than conventional adjuvant chemotherapy and hormone therapy. It is based on the reports [61–63] that women with trisomy 21 or Down syndrome (DS) have 10 to 25-fold less incidence of breast cancer compared to age-matched women with normal levels of chromosome 21. The endogenous antiangiogenic factor Endostatin is produced by genes located on chromosome 21. In view of the findings of the algorithm reported here, this suggests that before surgery to remove a newly diagnosed breast cancer, Endostatin should be increased to DS levels and kept at that high level indefinitely. Since persons with DS have no particular wound healing problems [64], this therapy will not interfere with recovery after surgery and should keep any micrometastases dormant as long as the therapy is continued [65]. It is non-toxic and drug resistance apparently does not develop, while that cannot be said for chemotherapy and hormone therapy. This could be considered the breast cancer analog to the colon cancer therapy as described previously.

There is a need for persons with the scientific discipline and perspective of experimental physics to work in cancer research. One interesting connection between detection of cancer and quantum physics has been pointed out by Badwe and Vaida [66] and I am sure there are more such overlaps yet to be discovered. There is much important work to be done in developing numerical models of cancer to guide scientists and physicians in research and in clinical settings. Other cancers need to be similarly analyzed—particularly melanoma, lung, prostate, and osteosarcoma. I would like to see 10 or more persons with PhD backgrounds in experimental physics doing cancer research. But they have to somehow or other be integrated into clinical and laboratory networks and to be sure to read the old literature [67].

As a sad end-note, Judah Folkman died in January 2008 at age 74 and Tim Browder died in March 2008 at age 51, both of sudden heart attacks. The week Dr. Folkman died, the lab personnel met at the regularly scheduled Friday morning meeting but instead of discussing angiogenesis, we shared memories. His chair was empty and the monogrammed lab coat that he always wore was draped over the back. During the next several hours, people just stood up and started talking about special memories of Judah Folkman. The picture that emerged from these often emotional comments was that Dr. Folkman was more than a physician, scientist, teacher, and mentor to us. He treated us like we were an extension of his family. He went to extreme lengths in our behalf. He would get the most skilled surgeon at Harvard to operate on someone’s child. He would visit someone in the hospital or he would call anywhere in the world to get something done or inquire about someone’s ill parent. Up until a few years ago, his home phone number was listed in the Boston public directory. Someone commented that Dr. Folkman was the only person he knew who had no hobbies. He was totally dedicated to helping others. Every evening he would call 10 of the cancer patients who called his office that day. Occasionally Judah Folkman spoke about retirement. But first he had a list of things he wanted to accomplish. He never told us what they were - and I have often wondered about that - but I am sure at least some are unfulfilled.

Tim Browder had not been seen around the lab for perhaps the past 5 years so only a few of us “old-timers” knew him. Tim was a dedicated and brilliant scientist and will be long remembered for his major contribution demonstrating what came to be called metronomic chemotherapy.
